# KLF13 Restrains Dll4-Muscular Notch2 Axis to Improve Sarcopenia

**DOI:** 10.1093/geroni/igaf122.1283

**Published:** 2025-12-31

**Authors:** Zhen Liang

**Affiliations:** Peking University Shenzhen Hospital, Shenzhen, Guangdong, China (People’s Republic)

## Abstract

Krüppel-like factor 13 (KLF13), a central regulator of cellular energy metabolism, is highly expressed in skeletal muscle and implicated in the pathogenesis of several diseases. This study investigated the role of KLF13 in sarcopenia. In a dexamethasone-induced muscle atrophy mouse model, compared to the control group, the KLF13 knockout group had decreased overall muscle strength (1.77 ± 0.10 vs. 1.48 ± 0.16, P < 0.01), muscle weight (%) 76.0 ± 5.69 vs. 60.7 ± 7.23, P < 0.001 for gastrocnemius (Gas) and 75.8 ± 6.21 vs. 67.5 ± 5.01, P < 0.05] for tibialis anterior, and exhaustive running distance (m) (495.5 ± 64.8 vs. 315.5 ± 60.9, P < 0.05). KLF13 overexpression preserved muscle mass (Gas: 100 ± 6.38 vs. 120 ± 14.4, P < 0.01) and exhaustive running distance (423.8 ± 59.04 vs. 530.2 ± 77.45, P < 0.05) in an in vivo diabetes-induced skeletal muscle atrophy model. Clofoctol protected against dexamethasone-induced muscle atrophy. Myotubes treated with dexamethasone were aggravated by KLF13 knockout, but anti-atrophic effects were achieved by inducing KLF13 overexpression. Transcriptome analyses and luciferase reporter assays demonstrate that delta-like 4 (Dll4) was a novel target gene of KLF13. KLF13 suppressed Dll4 expression, leading to the inhibition of the Dll4-Notch2 axis and prevention of muscle atrophy. Dexamethasone inhibited KLF13 expression by inhibiting myogenic differentiation 1-mediated KLF13 transcriptional activation and promoting F-Box and WD repeat domain containing 7-mediated KLF13 ubiquitination. These findings provide novel insights into mechanisms underlying skeletal muscle atrophy and sarcopenia with potential therapeutic targets.

